# Thiolated Cellulose: A Dual-Acting Mucoadhesive and Permeation-Enhancing Polymer

**DOI:** 10.1021/acs.biomac.3c00577

**Published:** 2023-10-05

**Authors:** Gergely Kali, Bengi Özkahraman, Flavia Laffleur, Patrick Knoll, Richard Wibel, Katrin Zöller, Andreas Bernkop-Schnürch

**Affiliations:** † Center for Chemistry and Biomedicine, Department of Pharmaceutical Technology, Institute of Pharmacy, University of Innsbruck, Innrain 80-82, A-6020 Innsbruck, Austria; ‡ Department of Polymer Materials, Faculty of Engineering, Hitit University, 19030 Corum, Turkey

## Abstract

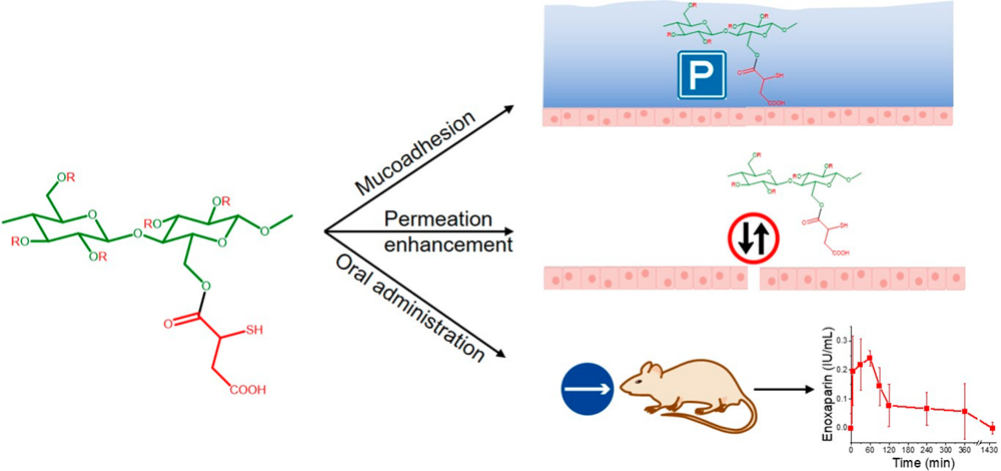

This study aims to design an anionic, thiolated cellulose derivative and to evaluate its mucoadhesive and permeation-enhancing properties utilizing enoxaparin as a model drug. 2-Mercaptosuccinic acid-modified cellulose (cellulose–mercaptosuccinate) was synthesized by the reaction of cellulose with *S*-acetylmercaptosuccinic anhydride. The chemical structure of the target compound was confirmed by FTIR and ^1^H NMR spectroscopy. The thiol content was determined by Ellman’s test. The conjugate exhibited 215.5 ± 25 μmol/g of thiol groups and 84 ± 16 μmol/g of disulfide bonds. Because of thiolation, mucoadhesion on porcine intestinal mucosa was 9.6-fold enhanced. The apparent permeability (*P*_app_) of the model dye Lucifer yellow was up to 2.2-fold improved by 0.5% cellulose–mercaptosuccinate on a Caco-2 cell monolayer. Enoxaparin permeation through rat intestinal mucosa increased 2.4-fold in the presence of 0.5% cellulose–mercaptosuccinate compared with the drug in buffer only. *In vivo* studies in rats showed an oral bioavailability of 8.98% using cellulose–mercaptosuccinate, which was 12.5-fold higher than that of the aqueous solution of the drug. Results of this study show that the modification of cellulose with 2-mercaptosuccinic acid provides mucoadhesive and permeation-enhancing properties, making this thiolated polymer an attractive excipient for oral drug delivery.

## Introduction

Polysaccharides, especially cellulose, are the most investigated excipients of oral drug delivery systems.^1^ Cellulose is a bio-based, biocompatible, and cheap polymer with low or no digestibility in the gastrointestinal tract. As a macromolecular excipient, cellulose is not absorbed and distributed in the systemic circulation and is thus generally recognized as safe (GRAS).^2^ The main limitations of cellulose in drug delivery are its low water solubility and the lack of interactions with the mucosal layer. In order to address these shortcomings, various cellulose derivatives, such as hydroxyethyl or carboxymethyl celluloses, exhibiting high swelling properties in aqueous media were designed, and some of them are widely used in marketed products.^3,4^ Mucoadhesive properties can be additionally introduced, especially by the thiolation of this polysaccharide.^5−8^ Because of thiol/disulfide exchange reactions with cysteine-rich subdomains of mucus glycoproteins, thiolated polymers, so-called thiomers, form disulfide bonds with the mucus layer and provide a prolonged mucosal residence time of drug delivery systems comprising them.^9−13^ Furthermore, thiomers show permeation-enhancing properties on mucosal membranes.^2,14^

In most cases, water-soluble cellulose derivatives, such as ethyl,^8^ hydroxyethyl,^5,6^ hydroxypropyl,^7^ or carboxymethyl^15,16^ celluloses, were thiolated via amide coupling or oxidation, followed by reductive amination with sulfhydryl ligands bearing a primary amine. The resulting cationic thiolated cellulose derivatives showed mucoadhesive and permeation-enhancing properties *in vitro*. The use of cationic substructures in drug delivery is mostly undesired because of the potential cytotoxicity of the positively charged groups. Recently, a novel thiolation method was described by our group in order to form anionic thiomers of lower toxicity.^17^ By covalent attachment of 2-mercaptosuccinic acid (MSA) to oligosaccharides, highly mucoadhesive thiomers were obtained.

Encouraged by these promising results, we intended to design a highly water-soluble thiolated cellulose derivative exhibiting high mucoadhesive and permeation-enhancing properties. For this reason, cellulose–mercaptosuccinate was synthesized and characterized in terms of thiol content, structure, solubility, and cytotoxicity. The mucoadhesive property of this cellulose derivative was determined by rheological studies using porcine intestinal mucus. The permeation-enhancing properties of this cellulose–mercaptosuccinate were quantified by *in vitro* and *ex vivo* studies using the water-soluble model dye Lucifer yellow and the model active pharmaceutical ingredient (API) enoxaparin. Furthermore, the potential of this thiolated cellulose derivative to improve the oral bioavailability of enoxaparin was evaluated in rats. Enoxaparin is a widely used anticoagulant^18^ which was chosen as a model drug because it shows poor oral bioavailability due to its high hydrophilicity and size. Strategies to increase the oral absorption of enoxaparin have already included co-administration with penetration enhancers^18−20^ or hydrophobic ion pairing.^21^

## Experimental Section

### Materials

Microcrystalline cellulose (Avicel-101) was provided by Meggle GmbH & Co. KG. 2-Mercaptosuccinic acid (MSA, 97%, Sigma-Aldrich), *N*,*N*-dimethylacetamide (≥99%, Sigma-Aldrich), dimethyl sulfoxide-*d*_6_ (DMSO-*d*_6_, 99.9 atom % D), and deuterium oxide (D_2_O, 99.9 atom % D) were all ordered from Sigma-Aldrich, Austria. Acetyl chloride (98%) was purchased from ACROS Organics, and Lucifer yellow CH dipotassium salt was purchased from VWR, Austria. Enoxaparin (Enoxaparin Becat 4000 IU (40 mg/0.4 mL), Rovi GmbH, Germany), 5,5′-dithiobis(2-nitrobenzoic acid) (Ellman’s reagent), Triton X-100, and minimum essential eagle medium (MEM) were obtained from Merck, Germany. All reagents were used as received.

### Cellulose Modifications

2-Mercaptosuccinic acid-modified cellulose (cellulose–mercaptosuccinate) was synthesized by esterifying native cellulose with *S*-acetylmercaptosuccinic anhydride.

In the first step, MSA (15 g, 0.1 mol) was mixed with acetyl chloride (24 mL, 0.34 mol) and refluxed for 5 h.^22^ The clear solution was concentrated using a rotary evaporator and precipitated into a cold diethyl ether. The formed product was filtered, further dispersed in diethyl ether, stirred, and centrifuged two times. After drying, *S*-acetylmercaptosuccinic anhydride was obtained as a white solid. Yield: 14.7 g, 84%. ^1^H NMR (DMSO-*d*_6_, 400 MHz) δ/ppm = 2.39 (s, 3H, C*H*_3_), 3.00 (m, 1H, C*H*_2_), 3.42 (m, 1H, C*H*_2_), 4.76 (m, 1H C*H*).

In a 100 mL round-bottom flask, 1 g of native cellulose (18.5 mmol of OH groups) and 12.8 g of *S*-acetylmercaptosuccinic anhydride (73.5 mmol, 4 equiv to OH) were dissolved in 35 mL of *N*,*N*-dimethylacetamide and stirred at 90 °C under a N_2_ atmosphere for 48 h. The reaction mixture was precipitated into diethyl ether, filtered, and dried in a vacuum. The solid was washed with a small amount of distilled water, dissolved in 1 M aqueous NaOH, filtered, and stirred for 2 h. The pH of the solution was set to 5.5 with 1 M HCl, and the solution was dialyzed against distilled water (Spectra/por6 dialysis membrane MWCO 3.5 kDa) at pH 6 for 3 days.

The target compound was obtained as a light brownish solid. Yield: 0.6080 g, 60%. ^1^H NMR (D_2_O, 400 MHz) δ/ppm = 2.19 (broad, s, 1H, C*H*_2_, mercaptosuccinate), 2.31 (broad, s, 1H, C*H*_2_, mercaptosuccinate), 3.40 (m, 1H C*H*, mercaptosuccinate). The remaining peaks between 2.5 and 4.75 ppm belong to the cellulose backbone.

Succinic acid-modified cellulose was synthesized as a control in order to investigate the effect of the thiol group. Other characterizations and measurements of this product have been discussed previously.^23−25^ In brief, 1 g of native cellulose (18.5 mmol of OH groups) and 7.4 g of succinic anhydride (73.5 mmol, 4 equiv to OH) were dissolved in 35 mL of *N*,*N*-dimethylacetamide in a 100 mL round-bottom flask and stirred at 90 °C under a N_2_ atmosphere for 48 h. The reaction mixture was precipitated into diethyl ether, filtered, and dried in a vacuum. The solid was dissolved in 0.01 M aqueous NaOH, filtered, and stirred for 2 h. The pH of the solution was set to 5.5 with 0.01 M HCl, and the solution was dialyzed against distilled water (Spectra/por6 dialysis membrane MWCO 3.5 kDa) at pH 6.

Modified cellulose was obtained as a whitish solid. Yield: 0.5703 g, 57%. ^1^H NMR (D_2_O, 400 MHz) δ/ppm = 2.19 (broad, s, 4H, C*H*_2_, succinate), 2.5 to 4.75 (broad, m, cellulose backbone).

### Chemical Characterizations

The thiol content of the target product was quantified using Ellman’s and disulfide bond tests.^26,27^ For Ellman’s test, 1 mg of modified cellulose was dissolved in 250 μL of 0.5 M phosphate buffer pH 8.0 (Ellman’s buffer), and 500 μL of 5,5′-dithiobis(2-nitrobenzoic acid) (Ellman’s reagent, with 0.3 mg/mL concentration in Ellman’s buffer) was added to the sample that was incubated for 2 h followed by centrifugation for 5 min. An aliquot of 100 μL was transferred to a 96-well plate, and absorption was measured at 450 nm using a microplate reader (Tecan infinite M200 spectrophotometer, Tecan Austria GmbH, Grödig, Austria). The amount of free thiol groups was calculated using a previously established calibration curve of l-cysteine under the same conditions. In order to quantify the disulfide content of the synthesized polymer, the above-described procedure was followed after reducing disulfide bonds via the addition of sodium borohydride (NaBH_4_, 4% aqueous solution) to the polymer solution.^27,28^ The experiments were performed in triplicate.

^1^H NMR measurements were performed on a “Mars” 400 MHz Avance 4 Neo spectrometer from Bruker Corporation (Billerica, MA, 400 MHz) in dimethyl sulfoxide-*d*_6_ (DMSO-*d*_6_) or deuterium oxide (D_2_O) solution.^27^

The Fourier-transform infrared (FTIR) spectra of native cellulose and cellulose–mercaptosuccinate were recorded by using a Bruker ALPHA FT-IR apparatus equipped with a Platinum ATR (attenuated total reflection) module. The FTIR spectra were normalized using the reference peak of C–O stretching vibrations at around 1000 cm^–1^.^27^

The solubility of the modified cellulose was determined in demineralized water according to the OECD method.^27,29^ Namely, 100 mg of cellulose–mercaptosuccinate was weighed into a 10 mL measuring flask, and 0.5 mL of demineralized water was added every 20 min while the flask was shaken at 25 °C. The solubility was determined from the amount of demineralized water needed to solubilize the polymer completely.

### Enzymatic Degradation of Cellulose–Mercaptosuccinate

The biodegradability of cellulose–mercaptosuccinate was investigated with preactivated lipase in a digestive medium consisting of 10 mM Tris buffer pH 7.0, also containing 5 mM CaCl_2_ and 150 mM NaCl, as described previously.^30^ Briefly, 1 g of lipase was added to 10 mL of digestive medium, followed by centrifugation at 13400 rpm at 4 °C for 20 min. Subsequently, the supernatant was stored at 4 °C for further use.^30^ For degradation studies, 60 mg of cellulose–mercaptosuccinate was dissolved in 3 mL of digestion medium with a pH of 7.0, having been adjusted with 0.1 M NaOH before adding 3 mL of lipase solution. The mixture was incubated at 37 °C for ester cleavage by lipase, resulting in a pH drop in the reaction mixture. In order to maintain the pH at 7.0, titration with 0.1 and 0.01 M aqueous NaOH was conducted at several predetermined time points for 300 min. Incubation was continued for up to 24 h to determine the MSA content in the sample. The amount of NaOH required was equal to the release of MSA.^30^ Native cellulose served as a negative control.

### Cytotoxicity Studies on the Caco-2 Cell Line

The cytotoxicity of native (microcrystalline) and modified cellulose was determined using the resazurin assay according to a previously described method.^27,31^ Briefly, Caco-2 cells were seeded in a 24-well plate at a density of 25,000 cells per well in minimum essential medium (MEM) supplemented with penicillin/streptomycin solution (100 units/0.1 mg/L) and 10% (v/v) fetal calf serum (FCS). Cells were incubated for 14 days at 37 °C under 5% CO_2_ and a 95% relative humidity environment. During the incubation period, the medium was replaced every 48 h. Hydrated samples of native and modified cellulose were prepared at concentrations of 0.1%, 0.25%, and 0.5% m/v in 25 mM HEPES buffered saline (HBS) pH 7.4. For the experiment, cells were washed twice with preheated HBS at 37 °C. Test solutions, pure HBS as the positive control, and 1% v/v Triton X-100 as the negative control, were added in triplicate to the cell culture plate in the volume of 0.5 mL/well and incubated at 37 °C in a 5% CO_2_ and 95% relative humidity environment for 4 and 24 h. After incubation, test solutions were removed, and cells were washed twice with preheated HBS at pH 7.4. An aliquot of 0.25 mL of a 2.2 mM resazurin solution was added to each well, and the cells were incubated again under the same conditions for 3 h. Thereafter, the supernatant’s fluorescence from each well was measured at 540 nm excitation wavelength and 590 nm emission wavelength.^27,28^ Cell viability was calculated by the following equation: cell viability [%] = {[average fluorescence of samples]/[average fluorescence of samples treated with 25 mM HEPES–268 mM glucose buffer pH 7.4]} × 100.

### Rheological Investigation

Viscoelastic properties of freshly isolated intestinal mucus mixed with native cellulose and cellulose–mercaptosuccinate were investigated. A local slaughterhouse provided the freshly excised porcine intestine. To isolate the mucus, the small intestine was cut into 10 cm pieces and opened longitudinally. The mucus was gently scraped off from the underlying tissue and purified subsequently.^32,33^ In brief, 1 g of mucus was diluted with 5 mL of 0.1 M NaCl, gently stirred for 1 h on ice, and centrifuged at 10400*g* (Sigma 3-18KS, Sigma Laborzentrifugen, Osterode am Harz, Germany) at 10 °C for 2 h. The supernatant and the granular material on the bottom of the centrifugation tube were discarded, and the procedure was repeated one more time. The purified mucus was homogenized before further use.

Experiments were performed utilizing a cone–plate combination rheometer^10,34^ (Haake Mars rheometer, 40/60, Thermo Electron GmbH, Karlsruhe, Germany; rotor: C35/1°, *D* = 35 mm) at a constant temperature of 37 °C, and the gap between cone and plate was 0.052 mm. Oscillatory stress sweep measurements within the region of linear viscoelasticity were performed with shear stress in the range 0.01–50.0 Pa while the frequency was kept constant at 1 Hz. Dynamic viscosity (η) values were measured for native and modified cellulose.^35^

For the rheological measurement, native cellulose and cellulose–mercaptosuccinate were dispersed or dissolved in 100 μL of 100 mM phosphate buffer pH 6.8 in a concentration of 0.3% (m/v). The suspensions/solutions and porcine mucus were mixed and homogenized in a ratio of 1:5. After incubation of 4 h at 37 °C without stirring, samples were analyzed, determining the viscoelastic characteristics.^17^

### Permeation-Enhancing Properties

#### *In Vitro* Permeation Studies on the Caco-2 Cell Monolayer

Permeation studies on the Caco-2 cell monolayer were performed as described previously.^14^ Caco-2 cells at 0.6 × 10^5^ cells/cm^2^ density were seeded onto 24-well plates with ThinCert inserts (pore size: 400 μm; Greiner Bio-One, Kremsmünster, Austria). Cells were cultured in MEM containing 20% FCS in an atmosphere of 5% CO_2_ and 95% humidity at 37 °C for 21 days, and the medium was renewed every 48 h. Only cell monolayers showing a transepithelial electrical resistance (TEER) above 500 Ω·cm^2^ were applied for permeation studies determined with an EVOM instrument. TEER values were also determined at the beginning of the measurement and after 3 and 24 h to confirm monolayer integrity. Before initiating the experiment, cells were washed with PBS buffer pH 6.8. Thereafter, 100 μL of MEM without FCS was transferred to the donor chamber, and 500 μL of the same medium was transferred to an acceptor chamber. After equilibration for half an hour, the medium in the donor chamber was replaced with 100 μL of 0.5% (m/v) native cellulose, cellulose–succinate, or cellulose–mercaptosuccinate containing Lucifer yellow in a final concentration of 0.05% (m/v) in MEM medium. Lucifer yellow solution (100 μL, 0.05% m/v) in MEM without FCS served as a control.^14^

At predetermined time points, aliquots of 100 μL were withdrawn from the acceptor chamber and replaced with the same volume of fresh preheated medium. At the end of the permeation experiment, test solutions in the donor and acceptor compartments were replaced with fresh prewarmed medium. The amount of dye permeated across the Caco-2 cell monolayer was quantified by fluorescent spectroscopy. The fluorescence of each samples was measured at 434 nm excitation wavelength and 540 nm emission wavelength.^14^

Apparent permeability coefficients (*P*_app_) for Lucifer yellow were calculated as described previously^36^ according to the equation
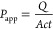
where *Q* is the total amount of Lucifer yellow dipotassium salt (μg) permeated through the monolayer, *A* is the diffusion area (1.13 cm^2^), *c* is the initial dye concentration (μg/cm^3^) in the donor chamber, and *t* is the time (s) of the permeation study. The permeation enhancement ratio (*R*) was determined as the ratio of *P*_app_ for the sample and the control.^14^

#### *In Vitro* Permeation Studies on Rat Intestinal Mucosa

For *in vitro* permeation studies, nonfasted Sprague–Dawley rats (250–300 g) were used. After the rats were sacrificed, the gut was removed and kept in normal saline. The distal ileum and jejunum segments were cut into strips of about 1.5 cm, opened longitudinally, and rinsed with a normal saline solution to free the luminal contents. Subsequently, the tissue was firmly fixed in Ussing chambers with a permeation surface area of 0.64 cm^2^ without removing the underlying muscle layer. The apical side of the chamber was filled with 1 mL of medium containing 5 mM KCl, 138 mM NaCl, and 10 mM glucose buffered with 10 mM HEPES pH 6.8, while the basolateral side of the chamber was also filled with 1 mL of this medium, additionally containing 1 mM MgCl_2_ and 2 mM CaCl_2_. These salts were used only in the acceptor site in order to prevent the complex formation of thiolated cellulose with the bivalent salts. A 5% CO_2_ and 95% O_2_-containing gas was used to purge each compartment to ensure oxygenation and agitation. Ussing chambers were equilibrated at 37 °C for 30 min to simulate the physiological intestinal conditions. The buffer medium of the apical side of the chamber was then replaced with a 0.5% (m/v) solution of native cellulose and cellulose–mercaptosuccinate mixed with enoxaparin in a concentration of 0.1% (m/v) in the apical site buffer for permeation studies.^14^ An aqueous, free drug solution at a concentration of 0.1% (m/v) served as a control. Every 30 min, 100 μL aliquots were taken from the basolateral chamber and replaced by the same volume of fresh medium prewarmed to 37 °C. The amount of enoxaparin that permeated the intestinal membrane was determined by Biophen Heparin Anti-Xa (2 Stages Heparin Assay) kit utilizing the manual method, determining the color intensity at 405 nm, with a microplate reader (Tecan infinite M200 spectrophotometer, Tecan Austria GmbH, Grödig, Austria). The apparent permeability coefficients (*P*_app_) and permeation enhancement ratio (*R*) were calculated as described above.^14^

### *In Vivo* Studies

All animal experiments were performed according to the Principles of Laboratory Animal Care as approved by the Animal Ethical Committee of Austria (BMBWF-66.008/0035-V/3*b*/2019). For the *in vivo* studies, Sprague–Dawley rats (average weight 250–300 g) were used.^14^ Rats were randomly divided into three groups: the first group (*n* = 3) for 500 μL of enoxaparin (0.1%) solution and the second group (*n* = 3) for 500 μL of a cellulose–mercaptosuccinate (0.5%)/enoxaparin (0.1%) solution. Both groups were dosed via oral gavage. The third group (*n* = 3) received enoxaparin in sterile 10 mM PBS pH 7.4 via i.v. injection. No food was supplied 2 h prior to dosing, but water was provided ad libitum. The first two groups were treated with a dose of 10 mg of enoxaparin per kilogram of body weight, while the third group received enoxaparin in a dose of 0.3 mg/kg. Blood samples (200 μL) were collected from the prominent lateral tail veins into citric acid-containing Eppendorf tubes at predetermined time points of 1, 30, 60, 90, 120, 240, and 360 min. To separate plasma, samples were centrifuged at 3000 rpm for 10 min, and enoxaparin content was quantified by Biophen Heparin Anti-Xa (2 Stages Heparin Assay) kit, as described above.

Pharmacokinetic analyses were applied to the plasma concentration–time data in a noncompartmental manner to obtain PK parameters of enoxaparin after the intravenous and oral administration of samples. AUC was determined with the linear trapezoidal rule, and absolute bioavailability was calculated by using the following equation:
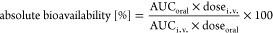


### Statistical Analysis

All experiments were performed in at least in triplicate. Data were analyzed with GraphPad Prism 5 (Graphpad Software, Inc., San Diego, CA). All data were represented as mean ± SD. Statistical comparisons were made using one-way ANOVA and two-way Bonferroni multiple comparisons test and *p* < 0.05 as the minimal level of significance.^28^

## Results and Discussion

### Synthesis and Characterization of Cellulose–Mercaptosuccinate

**Figure 1:**
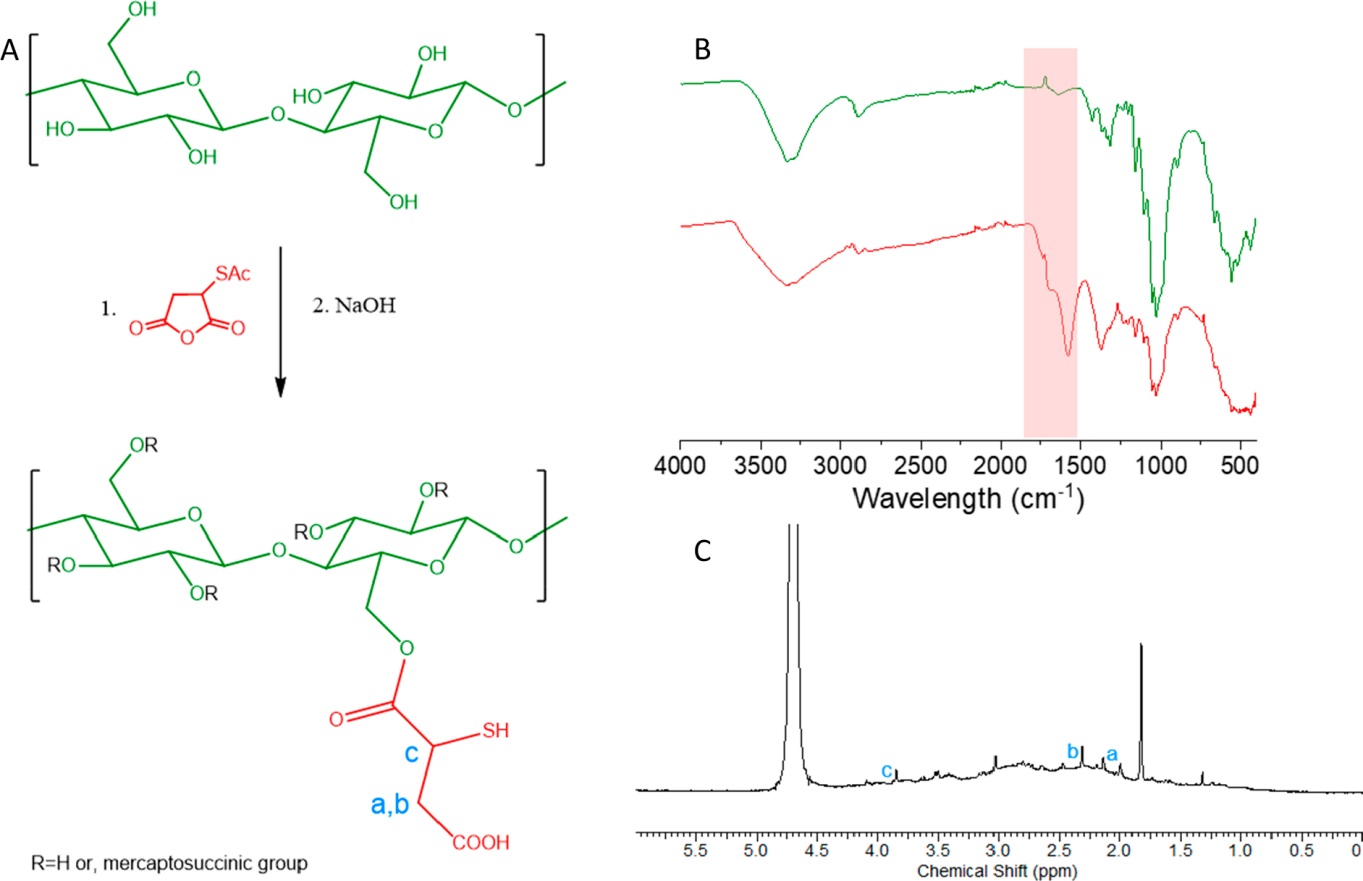
Proposed mechanism of the esterification of cellulose with *S*-acetylmercaptosuccinic anhydride (A), FTIR spectrum (B), and 400 MHz ^1^H NMR spectrum of the product in D_2_O (C).

Cellulose is a water-insoluble polysaccharide used for numerous pharmaceutical applications.^1^ Thiolated polymers are known as mucoadhesives and permeation enhancers.^2,14^ In order to combine these advantageous properties with hydration, the thiol and carboxylic acid-bearing ligand, 2-mercaptosuccinic acid, was conjugated to native cellulose as shown in Figure 1.

The ^1^H NMR spectrum of the product, presented in Figure 1, shows signals of cellulose between 1.5 and 4.5 ppm, including the H3–H6 protons between 2.5 and 3.8 ppm and some solvent peaks (H_2_O 4.75 ppm, DMAc 2.04 and 3.08 ppm, and diethyl ether 1.30 and 3.55 ppm) or acetyl group at 4.70 and 1.85 ppm. Also, some sharper peaks at 2.15, 2.30, and 3.90 ppm were observed, belonging to the −CH_2_– and ⟩CH– protons of the connected mercaptosuccinate moieties. Peaks slightly shifted below and above belong to the various modification sites, i.e., at C-2, C-3, and C-6 positions. FTIR measurements confirmed the derivatization of cellulose, as the strong peak of cellulose–mercaptosuccinate at 1741–1580 cm^–1^ for C=O carbonyl of the acid and ester appeared, indicating the formation of an ester after the reaction.

The amount of free thiols and disulfide bonds on modified cellulose was determined via Ellman’s test. The concentration of free thiols was 215.5 ± 25 μmol/g, while 84 ± 16 μmol/g disulfide bonds were found, indicating about 2.10 ± 0.2% modification of OH groups with mercaptosuccinate moieties. This thiol content is up to 13.4-fold higher than that described previously for thiolated cellulose derivatives.^15,16,37,38^

Cellulose–mercaptosuccinate solubility was determined in demineralized water.^29^ Results showed that esterification strongly enhanced the solubility in water, as 513.73 ± 2.15 g/L solubility was measured for the modified cellulose, while the native one was completely insoluble in aqueous media.

### Enzymatic Degradation of Cellulose–Mercaptosuccinate

**Figure 2:**
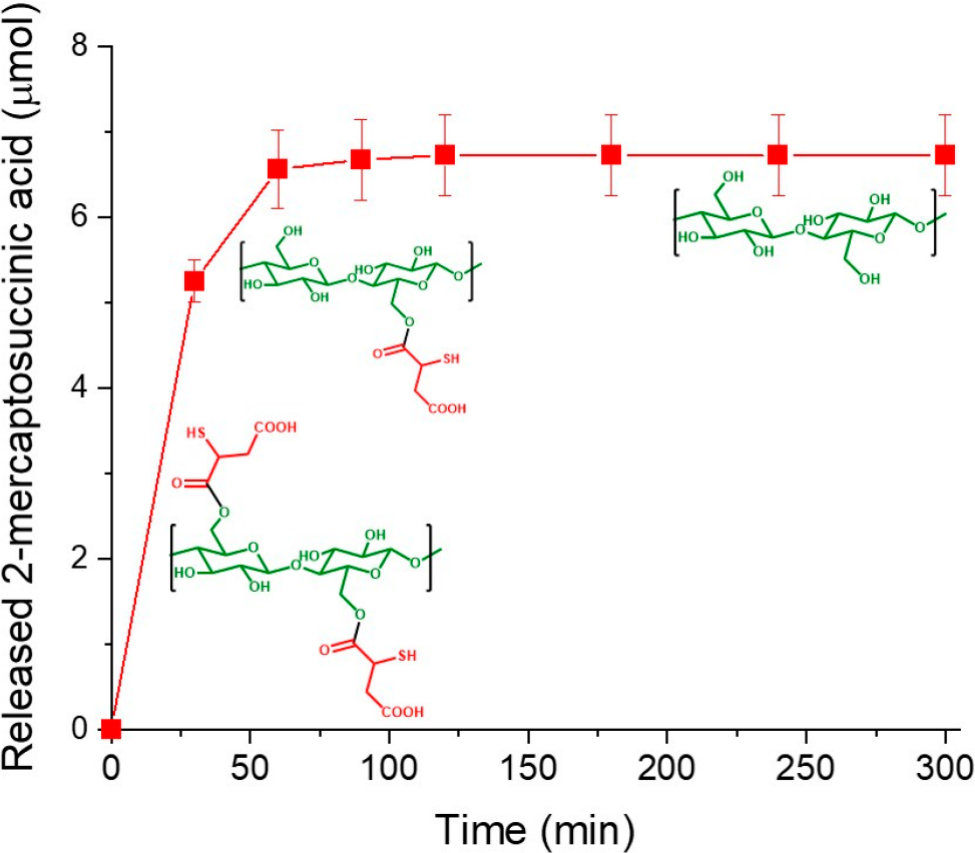
Enzymatic degradation studies of cellulose–mercaptosuccinate (10 mg/mL) using preactivated lipase (6250 U/mL) in a digestive medium (10 mM Tris buffer pH 7.0 with 5 mM CaCl_2_ and 150 mM NaCl). Data are presented as mean ± SD (*n* = 3).

Biodegradation of new polymeric excipients in drug delivery is a vital feature. Therefore, it was particularly important to investigate the lipase-catalyzed enzymatic degradation of the formed cellulose derivative. The results of enzymatic degradation studies are depicted in Figure 2. A rapid mercaptosuccinic acid release was observed in the first 30 min. After this initial 10.5 mmol/h burst release, the degradation slowed, releasing 1.4 mmol/h. This lipase-catalyzed ester hydrolysis presumably followed a Michaelis–Menten kinetic and was also observed for ester-modified hydroxyethyl cellulose^30^ and triacylglycerol or palm oil.^39,40^ After 2 h of reaction, the degradation was completed, and no further ester bond decomposition was detected. The determined amount of liberated ester (∼230 μmol/g) is close to the amount of thiols determined by Ellman’s test (∼299.5 μmol/g). Therefore, it can be assumed that the entire amount of mercaptosuccinic acid substructures cleaved off of the cellulose backbone.

### Cytotoxicity Studies

**Figure 3:**
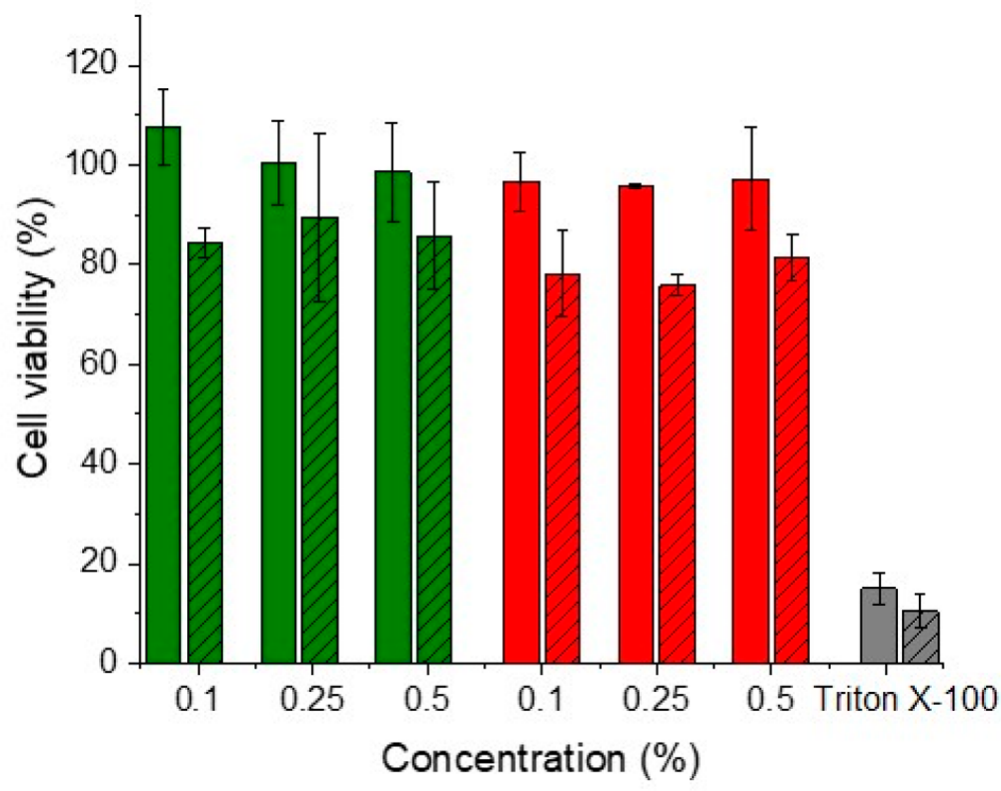
Cell viability of Caco-2 cells after 4 h (empty columns) and 24 h (columns with pattern) of incubation at 37 °C with native cellulose (green bars) and cellulose–mercaptosuccinate (red bars) at 0.1, 0.25, and 0.5% (m/v). The data are shown as mean ± SD (*n* = 3).

Cytotoxicity is the most crucial parameter for the design of novel excipients. The cytotoxic potential of the cellulose–mercaptosuccinate was investigated on Caco-2 cells at different concentrations and compared with that of native cellulose. Results of cytotoxicity studies are shown in Figure 3 after 4 and 24 h of incubation. In detail, there was no significant difference in the cytotoxicity of native and modified cellulose after 4 h, with overall cell viabilities of around 100% for all concentrations. After 24 h, the cell viability of native cellulose was above 80% for all tested concentrations, while slightly lower values were found for modified cellulose (between 75 and 81%). Excipients providing cell viability above 80% are generally considered safe.^41^ These results show that the formed new cellulose derivatives have no or a minor cytotoxic effect.

### Rheological Properties

**Figure 4:**
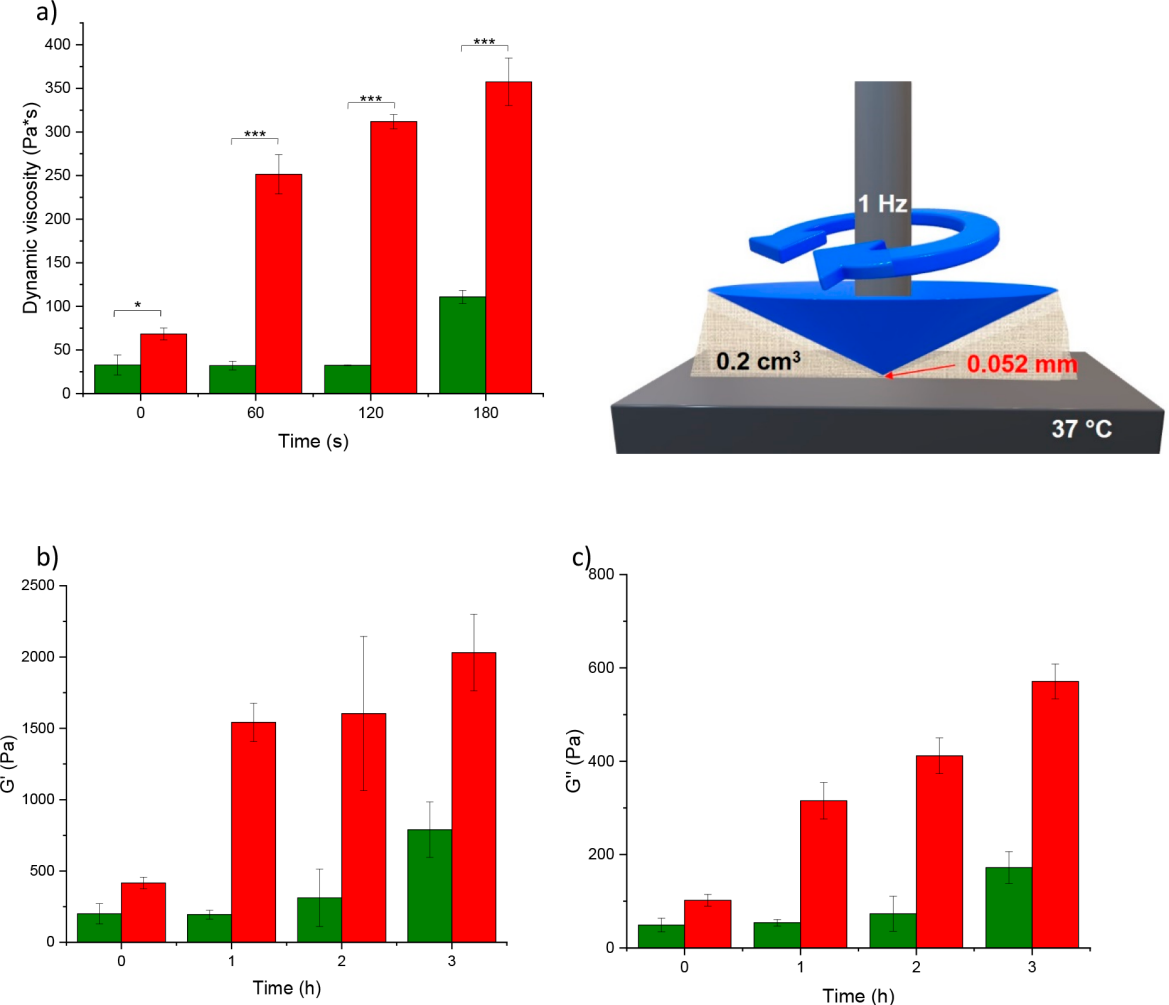
Rheological behavior of native cellulose (green bars) and cellulose–mercaptosuccinate (red bars) (2% m/v) porcine mucus mixture (1:5). Dynamic viscosity (η) (a), elastic modulus (*G*′) (b), and viscous modulus (*G*″) (c) were determined immediately after mixing (0 min) and after 1, 2, and 3 h of incubation at 37 °C. Indicated values are outlined as means ± SD (*n* ≥ 3) (***p < 0.001, *p < 0.05). Upper right: schematic representation of the rheometer.

Cysteine-rich mucus glycoproteins in the mucus are readily available for disulfide bond formation with thiol-bearing excipients. Thiol/disulfide exchange reactions lead to disulfide bonds between thiolated cellulose and mucus, providing a prolonged mucosal residence time for the thiomer. In order to investigate the availability of thiols in the cellulose–mercaptosuccinate structure for disulfide bond formation leading to cross-linking, the rheological properties of its mixture with freshly collected and purified porcine intestinal mucus were investigated using a cone plate rheometer. The schematic representation of the setup is depicted in Figure 4, right-hand upper side. The results were compared with those of native cellulose.

As shown in Figure 4, the viscosity of the modified cellulose–mucus mixture increased immediately after the addition and further improved over time. Compared with native cellulose, a 2.1-fold higher viscosity was detected even instantly after mixing. Viscosity increased further over time, and after 2 h, 9.6-fold higher viscosity was detected for cellulose–mercaptosuccinate compared to native. This difference at the given degree of modification suggests that probably not only thiol groups are responsible for mucoadhesion. Besides them, the carboxylic moieties on the cellulose backbone can form hydrogen bonds with mucus glycoproteins, contributing to mucoadhesion.

Additionally, the storage modulus (*G*′, elastic component), as well as the loss modulus (*G*″, viscous component), increased over time for the cellulose–mercaptosuccinate. The *G*′ and *G*″ values reached up to 2.6- and 3.3-fold enhancement compared to native cellulose, respectively, indicating an increasing number of cross-linking points in its mixture with mucus.

### Permeation Enhancement

Thiolated oligomers and polymers are known as permeation enhancers,^2,14^ inhibiting protein tyrosine phosphatase (PTP) via the formation of disulfide bonds. This enzyme dephosphorylates occludin, a transmembrane protein of tight junctions. Generally, phosphorylated occludin opens tight junctions while the dephosphorylated version closes them.^2^ By the inhibition of PTP, dephosphorylation of this protein and, consequently, closing of tight junctions cannot take place. The permeation of the water-soluble fluorescent dye Lucifer yellow alone and in mixtures with native and modified cellulose was investigated on a Caco-2 cell monolayer. Because of the mucoadhesive properties of the cellulose–mercaptosuccinate, longer permeation times than the small intestinal residence time of 182 ± 69 min^42^ were tested.

**Figure 5:**
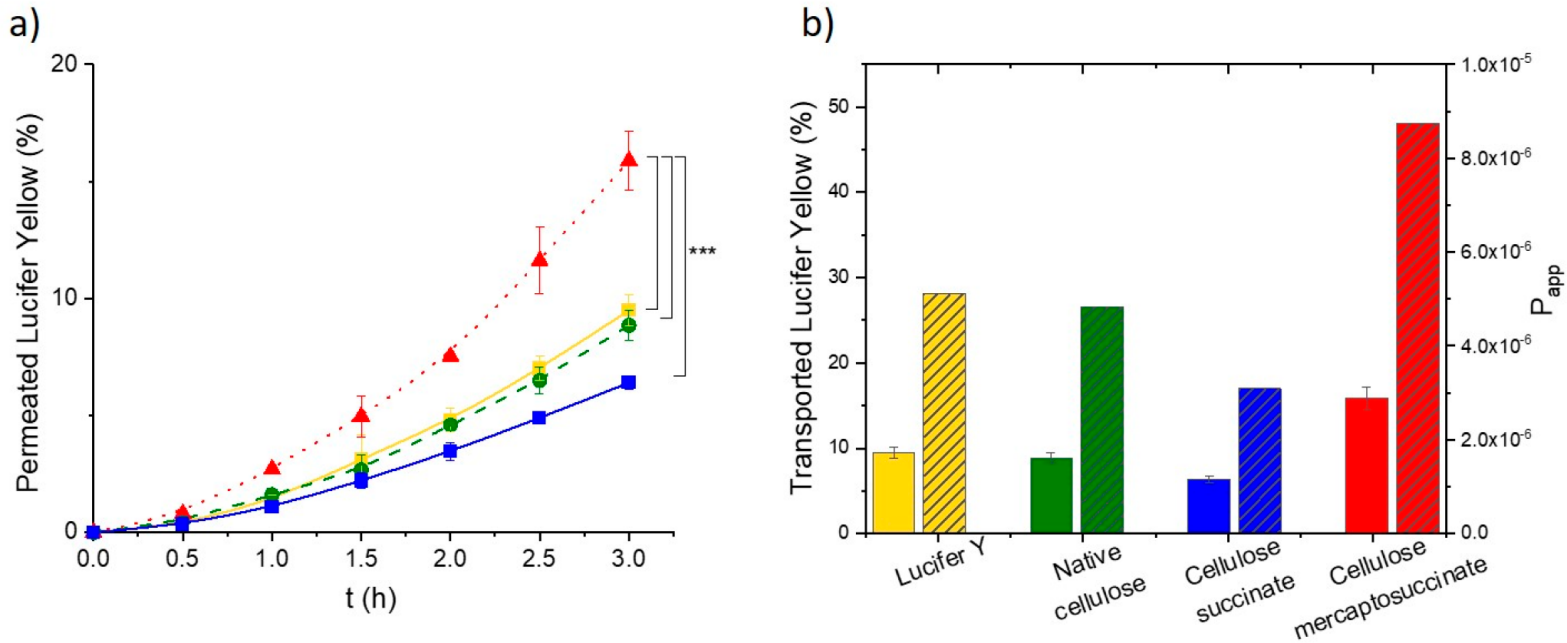
Permeation enhancing effect of cellulose–mercaptosuccinate on Lucifer yellow diffusion on Caco-2 cells. The time dependency of permeation of the dye on Caco-2 cells as the percentage of the initial concentration of Lucifer yellow. Lucifer yellow in buffer (yellow ▼), with native cellulose (green ●), cellulose–succinate (blue ■), and cellulose–mercaptosuccinate (red ▲) (a) and the amount of permeated fluorescent dye after 24 h (columns with no pattern) as the percentage of the initial concentration of Lucifer yellow and the apparent permeability constants (columns with striped pattern) for Lucifer yellow in buffer (yellow), with native cellulose (green), cellulose–succinate (blue), and cellulose–mercaptosuccinate (red) (b). All the results are shown as mean ± SD, *n* = 3 (***p < 0.001).

The results of the permeation studies using 0.05% (m/v) dye and 0.5% (m/v) native cellulose, cellulose–succinate, and cellulose–mercaptosuccinate are shown in Figure 5. Cellulose–succinate served as a control and confirmed the importance of the thiol group for the permeation enhancement. Native and succinic acid-modified cellulose did not alter the permeation behavior of Lucifer yellow. Compared to those two polymers, cellulose–mercaptosuccinate increased the amount of permeated dye to 15.90%, and the *P*_app_ was 1.8-fold higher than that of the dye alone.

Already after 1 h, thiolated cellulose significantly enhanced the amount of permeated dye compared to the other cellulose derivatives (Figure 5b). After 3 h, this increase in the amount of permeated dye was even more pronounced, reaching up to a 2.5-fold higher value than the other samples.

Moreover, the mucoadhesive properties of cellulose–mercaptosuccinate may further increase the residence time at the absorption site and consequently provide a prolonged time that is available for permeation. Therefore, the amount of permeated dye was measured for a prolonged period of 24 h. The differences became more significant as 2.2-, 3.3-, and 2.9-fold enhancements could be reached.

**Figure 6:**
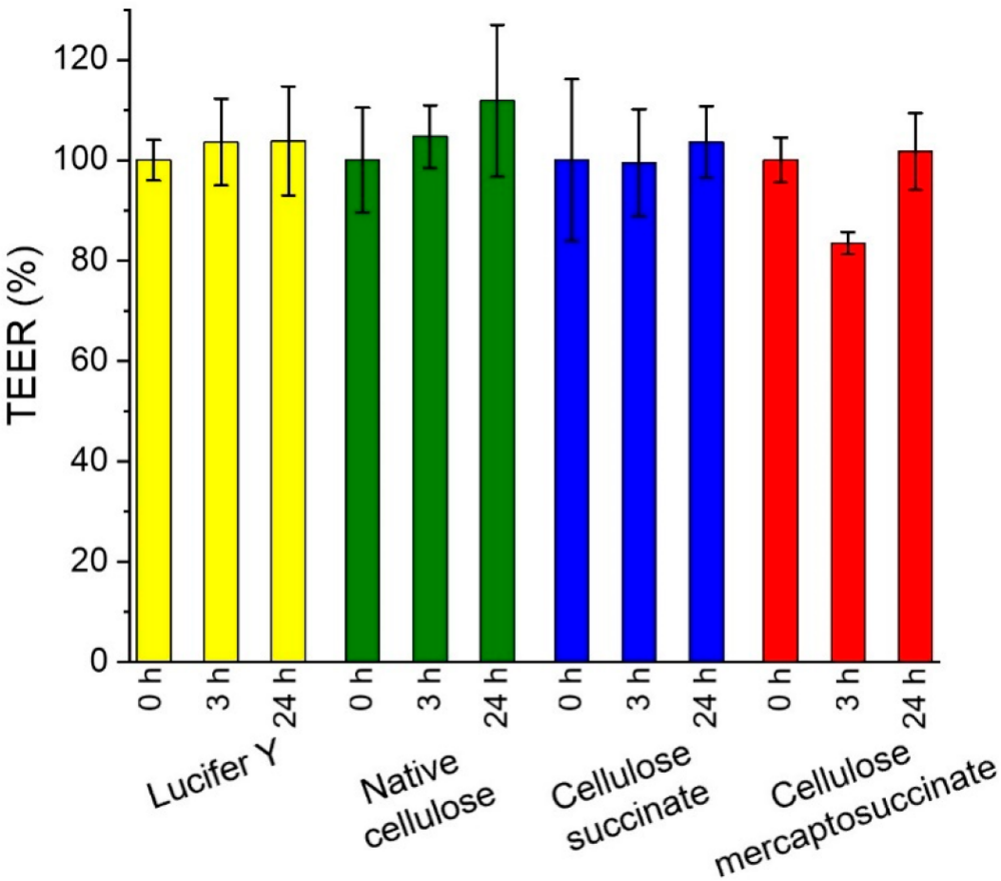
TEER of test solutions with Lucifer yellow (yellow), native cellulose (green), and cellulose–mercaptosuccinate (red) before initiating the permeation study (0 h), at the end of the permeation study (3 h), and after 24 h having replaced test solutions after the permeation study with buffer only. Results are presented as mean ± SD, *n* = 3.

Furthermore, transepithelial electrical resistance (TEER) values were determined as a measure of the tight junction opening. The TEER values, presented in Figure 6, did not alter significantly in the presence of native cellulose but decreased by almost 20% in the presence of cellulose–mercaptosuccinate compared to the free dye, confirming the opening of tight junctions. After 24 h, the cells were washed with MEM, and the TEER values returned to their initial values, proving the reversibility of tight junction opening.

**Figure 7:**
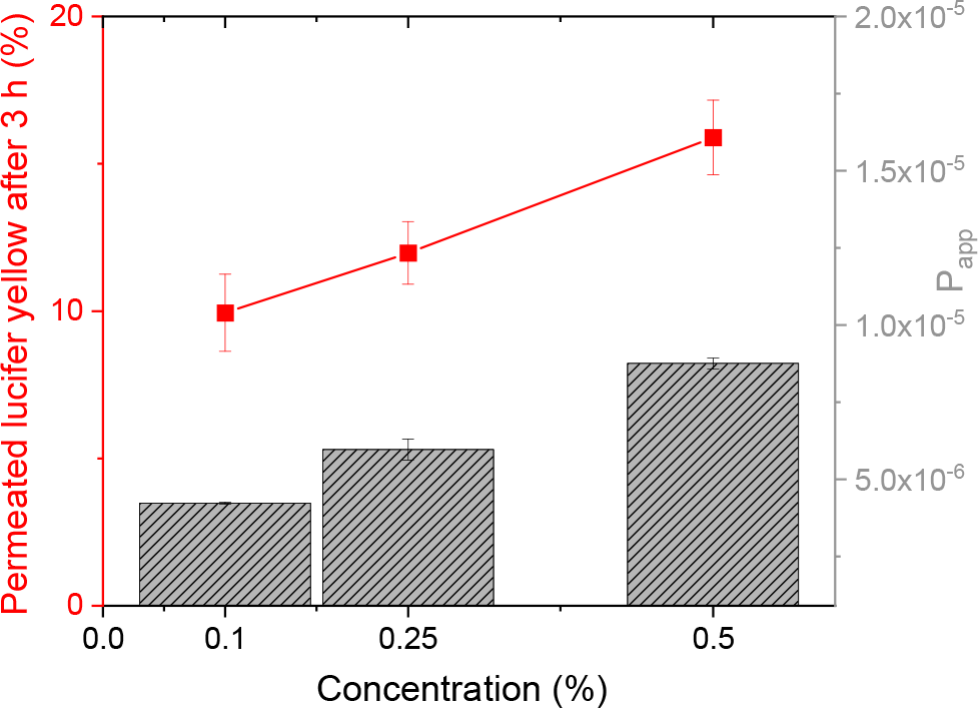
Concentration dependency of the permeation enhancement of cellulose–mercaptosuccinate on the diffusion of Lucifer yellow across Caco-2 cells. Results show the permeation of Lucifer yellow across Caco-2 cells at the indicated concentrations of thiolated cellulose after 3 h. All the results are shown as mean ± SD, *n* = 3.

The concentration dependency of the permeation enhancement was investigated at concentrations of 0.1, 0.25, and 0.5% (m/v) cellulose–mercaptosuccinate at the 3 h time point, and the results are shown in Figure 7. The concentration of cellulose–mercaptosuccinate greatly influenced the permeability of the cell monolayer, as the permeated amount of Lucifer yellow as well as the *P*_app_ increased with the polymer concentration. In detail, as the cellulose–mercaptosuccinate concentration increased from 0.1 to 0.5% (m/v), the amount of permeated dye and the *P*_app_ increased 1.6- and 2.1-fold, respectively.

**Figure 8:**
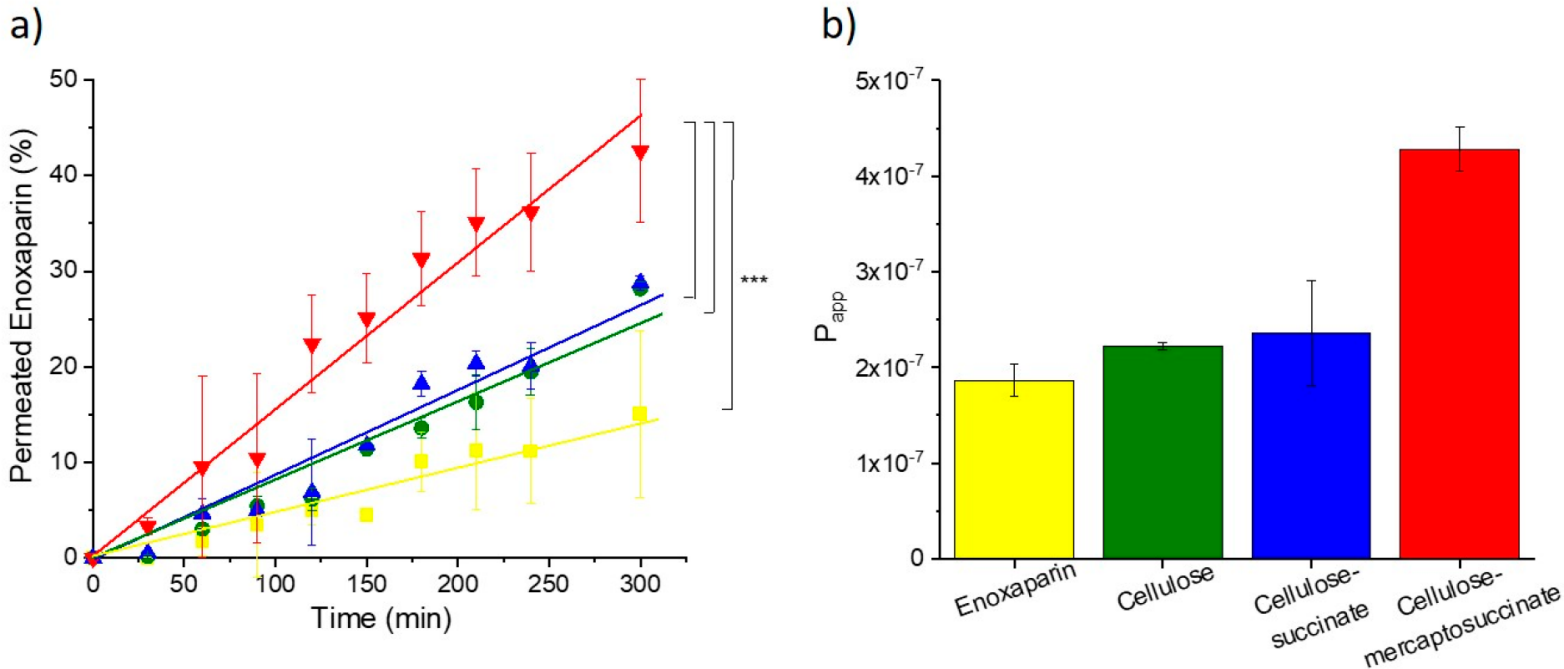
Permeation of 0.1% (m/v) enoxaparin across gut mucosa of nonfasting Sprague–Dawley rats. Drug solution without polymer (yellow) and in combination with 0.5% (m/v) native cellulose (green), 0.5% (m/v) cellulose–succinate (blue), and 0.5% (m/v) cellulose–mercaptosuccinate (red). Permeation data (a) are shown as percent of the total dose and apparent permeability constants (b) after 4 h. All the results are presented as the mean ± SD, *n* = 3 (***P < 0.001).

Permeation studies on freshly excised rat gut were performed in Ussing chambers using enoxaparin as a hydrophilic macromolecular model drug. Only a slight difference was found between enoxaparin solutions with and without native cellulose or cellulose–succinate. Between 3.04 and 3.84% of enoxaparin was detected on the basolateral side in the cases of free drug, cellulose, and cellulose–succinate, as shown in Figure 8. Also, the *P*_app_ values remained low, between 1.86 × 10^–7^ and 2.36 × 10^–7^. Contrarily, by the addition of cellulose–mercaptosuccinate, the amount of permeated API, as well as the *P*_app_, increased significantly by 2.3- and 2.4-fold compared to the control, respectively. This is a significant enhancement of permeation compared not only to native cellulose or cellulose succinate but also to thiolated hydroxyethylcellulose, described in a previous study, where 1.9-fold enhancement was reached.^37^

### *In Vivo* Studies

**Figure 9:**
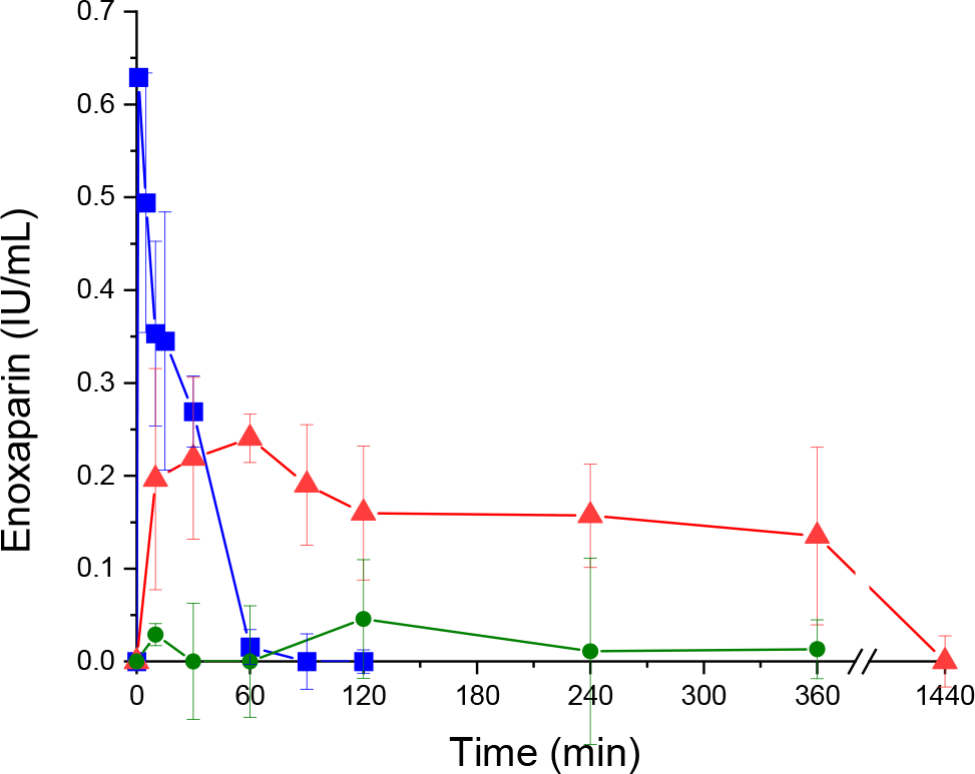
Concentration–time profile of enoxaparin in plasma after intravenous administration of aqueous enoxaparin solution (0.2 mg/kg) (blue ■) and oral administration of aqueous enoxaparin solution (10 mg/kg) without (green ●) and with 0.5% (m/v) cellulose–mercaptosuccinate (red ▲) to rats. Blood was collected from all cohorts at the indicated time points. Enoxaparin was quantified by Biophen Heparin Anti-Xa (2 Stages Heparin Assay) kit. All indicated values are means ± SD, *n* = 3.

*In vivo* studies with enoxaparin as a model drug were performed on Sprague–Dawley rats. To enhance intestinal absorption and oral bioavailability of enoxaparin, a cellulose–mercaptosuccinate/drug solution was administered orally. Intravenous and oral administration of enoxaparin solutions without the polymer were used as control. Plasma concentration–time profiles of all samples administered intravenously and orally are depicted in Figure 9, while the pharmacokinetic parameters are summarized in Table 1.

**Table 1:** Pharmacokinetic Parameters of Intravenously and Orally Administered Enoxaparin with and without Cellulose–Mercaptosuccinate

	dose (mg/kg)	AUC_0–8_ (μg/mL h)	*C*_max_ (IU/mL)	*T*_max_ (h)	bioavailability (%)
enoxaparin i.v.	0.2	15.22	0.62936	0	
enoxaparin oral	10	5.45	0.04576	2	0.72
enoxaparin + cellulose–mercaptosuccinate oral	10	68.36	0.24058	1	8.98

The oral administration of enoxaparin resulted in low *c*_max_ after 2 h and also low bioavailability around 0.72%, as shown in Table 1, as was expected for a poorly absorbable drug in the small intestine. In contrast, by the co-administration of cellulose–mercaptosuccinate, the *c*_max_ increased 4.3-fold, while the absolute bioavailability reached 8.98%, which is 12.5-fold higher than that of an orally administered aqueous enoxaparin solution serving as a control. Also, even after 6 h, an increased plasma concentration of enoxaparin was detected for the cellulose–mercaptosuccinate/drug system compared to orally or parentally administered pure enoxaparin solutions. This enhancement in bioavailability is due to the sulfhydryl groups of the cellulose–mercaptosuccinate, presenting mucoadhesive and permeation-enhancing properties that support drug absorption in the small intestine.

## Conclusion

Cellulose–mercaptosuccinate was synthesized and used as a mucoadhesive and permeation-enhancing excipient, and its application potential was confirmed by a model dye and hydrophilic macromolecular drug with low bioavailability for the first time. Cellulose–mercaptosuccinate showed an enhanced interaction with porcine intestinal mucus. Compared to native cellulose, the permeation of a dye or drug through the Caco-2 cell monolayer and rat gut was highly increased by the addition of cellulose–mercaptosuccinate. The apparent permeability constant increased up to 2.4-fold in the case of cellulose–mercaptosuccinate addition compared to free dye/drug or with its mixture with native cellulose. *In vivo* studies resulted in a 12.5-fold higher oral bioavailability in the case of the modified cellulose excipient compared to the free drug solution. The results highlight that this highly water-soluble, thiolated cellulose derivative could be a useful excipient to increase the gastrointestinal absorption of otherwise poorly adsorbed drugs.
